# Clinical Profile and Outcome of Esophageal Button Battery Ingestion in Children: An 8-Year Retrospective Case Series

**DOI:** 10.1155/2019/3752645

**Published:** 2019-12-01

**Authors:** Mustafa Erman Dörterler

**Affiliations:** Harran University Faculty of Medicine, Department of Pediatric Surgery, Şanlıurfa, Turkey

## Abstract

**Objective:**

To present the clinical profile and outcomes of esophageal button battery ingestion cases treated at our institution over an 8-year period.

**Methods:**

A total of 17 children who presented after ingesting a button battery and were treated at a tertiary care clinic over an 8-year period were included in this retrospective case series study. Data on patient demographics and esophageal location of the battery, time from ingestion to admission, symptoms, grade of mucosal injury, size of the battery, management, complications, and follow-up outcome were recorded.

**Results:**

Median age was 29 months (range, 2–99 months). Boys comprised (*n*=11, 64.7%) of the study population. The most common location was the proximal esophagus (*n*=10, 58.8%). The median time from ingestion to admission was 6 h (range, 3–24 h). Hypersalivation alone (*n*=6, 35.3%) or together with vomiting (*n*=5, 29.4%) was the most common symptom. Grade IIA mucosal injury was noted in six (*n*=6, 35.3%) patients. The diameter of the battery was a median of 18.0 mm (range, 14–22 mm). We did not observe any correlation between the size of the battery and the grade of the injury. Early postoperative complications were encountered in one patient (*n*=1, 5.8%) and late postoperative complications were noted in eight patients (*n*=8, 47.1%) which required further esophageal dilatations, and follow-up revealed normal findings in eight patients (*n*=8, 47.1%) and mortality occurred in one patient.

**Conclusion:**

The current case series study describing the clinical profiles and outcomes of 17 children who had ingested an esophageal button battery revealed male predominance, young patient age, and admission after a median of 6 h (3–24 h) of ingestion with nonspecific symptoms. Our findings confirm the success of rigid endoscopy to remove esophageal button batteries and indicate the likelihood of severe complications after removal.

## 1. Introduction

Ingestion of a button battery by children is considered an absolute surgical emergency and a dangerous and challenging form of foreign body ingestion that requires a rapid diagnosis and urgent removal [1–3].

An increase in button battery ingestion rates in children has occurred in recent years due to the spread of home multimedia devices that use larger batteries that may lead to life-threatening consequences, such as perforation or fistula, particularly in the case of esophageal impaction, even after removal of the battery from the esophagus [1–5].

Ingesting a button battery carries the risk of rapidly progressing and potentially life-threatening damage to the esophagus, due to electrical injuries (flow of electrical current from the positive to negative terminals of the battery bridged by the mucosa), mechanical injuries (pressure necrosis by mucosal compression), and caustic injuries (leakage of alkaline electrolytes and coagulative necrosis) [2, 3, 5–9].

Given experimental and clinical data that show that coagulative necrosis starts within 15 min of battery-esophageal contact [10] and that major corrosive injury begins within hours of ingestion [11]; the urgent endoscopic removal of a battery from the esophagus is a well-accepted approach [1].

This retrospective case series study was designed to present the clinical profiles and outcomes of esophageal button battery ingestion cases treated at our institution over an 8-year period.

## 2. Materials and Methods

A total of 17 children who presented to a tertiary care clinic after ingesting a button battery between January 2011 and December 2018 were included in this retrospective case series study. Data on patient demographics (age and gender), size, and esophageal location of the battery, time from ingestion to admission, symptoms on admission, witnessing of the event, grade of mucosal injury according to the Zargar classification [12], management, complications, and follow-up outcome were recorded for each case.

Posteroanterior and lateral chest radiographs were taken in all cases. Emergency rigid esophagoscopy was performed under general anesthesia for all patients. Anesthesia was induced with 2 mg/kg propofol or 3 mg/kg pentothal injection, and 1 *μ*g/kg remifentanil was administered as narcotic analgesia. The anesthesia was maintained with 2–4% sevoflurane in a 50% O_2_/50% air mixture, and the operation continued under controlled ventilation. The batteries were removed using rigid esophagoscopy and a foreign body forceps. Standard monitoring was applied, including electrocardiography and measurements of systolic and diastolic arterial blood pressures, heart rate, and peripheral oxygen saturation. Parenteral ampicillin-sulbactam (four doses per day at 100–200 mg/kg) was started as an empirical antibiotic in all cases. A postoperative evaluation was made with a chest X-ray and a physical examination. All children were monitored in the hospital for at least 24 h following the procedure.

## 3. Results

### 3.1. Overall Characteristics

The median age of the patients was 29 months (range, 2–99 months). Boys comprised (*n*=11, 64.7%) of the study population. The median time from ingestion to admission was 6 h (range, 3–24 h) in 12 cases and unknown in four cases, and one case was admitted 40 days after battery ingestion. Overall, in eight (*n*=8, 47.1%) cases, the ingestion was not witnessed. Hypersalivation alone or together with vomiting were the most common admission symptoms, as noted in six (*n*=6, 35.3%) and five (*n*=5, 29.4%) cases, respectively. The most common location for the ingested button battery was the proximal esophagus (*n*=10, 58.8%), followed by midesophagus (*n*=3, 17.6%) and the distal esophagus (*n*=4, 23.5%). The median battery diameter was 18.0 mm (range, 14–22 mm). We found no correlation between the battery size and mucosal damage. We believe that this may be due to our small sample size. Grade IIA mucosal injury was noted in 6 (*n*=6, 35.3%) patients. Postoperative complications were noted in three patients, follow-up revealed normal findings in nine (*n*=9, 52.9%) patients, and dilatation was required in seven patients (*n*=7, 41.2%) The diameter of the battery was a median of 18.0 mm (range, 14–22 mm). Early postoperative complications were encountered in one patient (*n*=1, 17.6%), and late postoperative complications were noted in eight patients (*n*=8, 41.2%), which required further esophageal dilatations. We had one vocal cord paralysis as early complication, and late complications comprised of two tracheaesophageal fistula and seven esophageal strictures. Mortality occurred in one patient with tracheaesophageal fistula which led to pulmonary infection and subsequent sepsis (Table 1).

### 3.2. Cases with Complications and the Need for Dilatation

**Figure 1:**
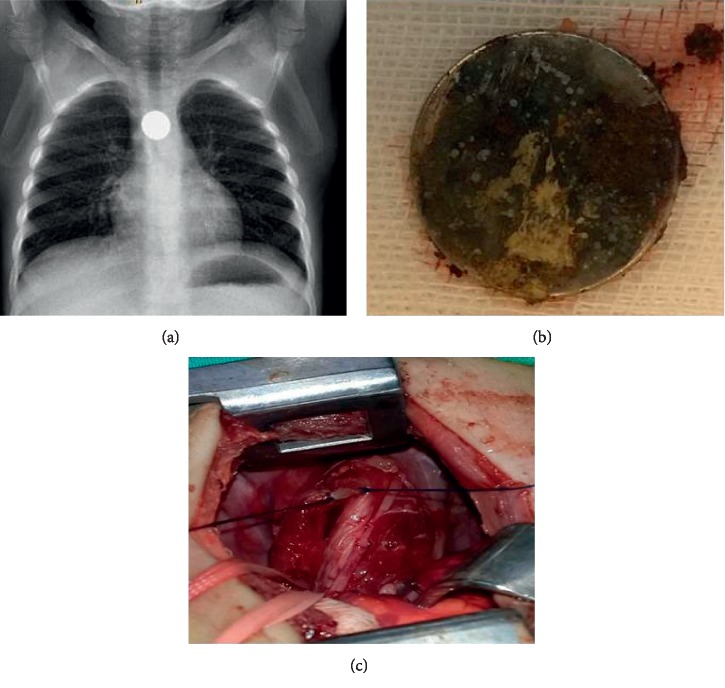
Images from a 2-month-old boy who was admitted to the hospital 40 days after ingesting a button battery. (a) Anteroposterior chest X-ray with double-contour aspect of the battery lodged in the proximal esophagus. (b) Battery after endoscopic removal. (c) Surgery due to development of a tracheoesophageal fistula.

A 2-month-old boy with a recurrent pulmonary infection and fever was admitted 40 days after ingesting a battery. The battery was located in the proximal esophagus, and surgery was required due to development of a tracheoesophageal fistula (Figure 1 and Table 2).

A 39-month-old boy was admitted 24 h after ingesting a battery with a complaint of dysphagia. The battery was located in the midesophagus, and the mucosal injury was grade IIIA. The patient developed unilateral vocal cord paralysis and required a dilatation intervention (Table 2).

A 2-month-old girl was admitted 5 h after ingesting a battery with a complaint of hypersalivation. The battery (14 mm) was located in the proximal esophagus, and the patient developed a tracheoesophageal fistula followed by subsequent sepsis and died 4 days later (Table 2).

None of the grade 0 or grade I cases required dilatation, whereas two (33.3%) of six cases with grade IIA, one of two (50.0%) cases with grade IIB, and all cases (two for each) with grades IIIA and III B required dilatation (Table 2).

Overall, dilatation was needed in two (33.3%) of six girls and five (45.5%) of 11 boys (Table 2).

### 3.3. Delay in Hospital Admission

Four cases with grade 0-I mucosal injuries were admitted to the hospital at a median of 5 h (range, 3–6 h) after ingestion. Six cases with grade IIA-B injury were admitted to the hospital at a median of 5.5 h (range, 4–8 h) after ingestion, whereas three cases with grade IIIA-B injury were admitted to the hospital at a median of 24 h (range, 6–24 h) after ingestion (Table 2).

Two cases with a midesophageal location of the battery were admitted to the hospital at 24 h after ingestion, whereas those with proximal or distally located batteries were admitted to the hospital at 3–8 h after ingestion (Table 2).

Our small group showed us that any delay could lead to serious complications, even to death. We could speculate that an interventional latency in Grade IIA injury that developed even after 5 hours needed future esophageal dilatations and such cases should be dealt with utmost speed.

## 4. Discussion

In the present case series, 64.7% of the children were boys (age 2–99 months), and the initial symptoms were nonspecific. This is consistent with the clinical profile of reported battery ingestion cases in the literature, including male predominance (58.7–84.6%) [2, 13], very young age [2, 14], and absence of specific clinical signs [2, 15, 16].

In a case series of 16 children who ingested a button battery, vomiting (33.3%), swallowing and/or feeding problems (27.8%), and fever (27.8%) were the most common symptoms [8]. In another case series of 26 children who ingested a button battery, the initial clinical signs at ingestion were vomiting (38.5%), fever (26.9%), and hypersialorrhea (26.9%) in most cases and chest pain, dysphagia, cough, or dysphonia were less frequently noted symptoms [2]. In yet another case series of eight children who ingested a button battery, the presenting clinical symptoms were dysphagia, coughing, vomiting, hypersalivation, fever, poor appetite, and recurrent pulmonary infection [9, 17–20]. The symptom profile observed in our population was in accordance with these published case series, which revealed that hypersalivation or dysphagia with or without vomiting, coughing, and recurrent pulmonary infection and fever were the most common presenting symptoms.

The nonspecificity of the initial presentation is important, given that it has been included among the factors leading to a delayed diagnosis together with failure to detect the battery on an X-ray and lack of awareness of the seriousness of the condition by the initial care team [2, 21–23]. In addition, given that in nearly half of the cases in our series the ingestion event was unwitnessed, more than 50% of serious outcomes due to button battery ingestion likely occur after unwitnessed ingestion, due to the nonspecific character of the symptoms coupled with the high likelihood of a delay in recognition and diagnosis [14, 17].

Button batteries are the second most frequently ingested foreign body after coins [24]. It is crucial to differentiate coin ingestion from button battery ingestion because of the severity of the complications resulting from button battery ingestion [1, 9]. Accordingly, both anteroposterior and lateral chest X-rays should be taken in all children with a suspected diagnosis of a foreign body ingestion; demonstration of the halo sign in anterior chest X-ray and the step-off sign in lateral chest X-ray film are diagnostic of button battery ingestion [1, 9, 25].

However, morbidity and mortality associated with ingesting a button battery are not strictly limited to vascular injury and bleeding events, but also include serious complications likely to develop after removing the battery, such as esophageal-tracheal fistulas, esophageal perforations, esophageal stenosis, vocal cord paralysis, pneumothorax, aspiration pneumonia, spondylodiscitis, esophageal-aortal fistulas, and respiratory and circulatory failure [1, 8]. Thus, follow-up care for patients after removing a button battery is considered essential to assess midterm complications, such as bleeding, and long-term sequelae, such as stricture formation, which should be promptly managed via endoscopic dilation [1].

In a reported series of 13 cases with severe esophageal injury from ingesting a battery, four (30.8%) cases resulted in an esophageal perforation, three (23.1%) developed an esophageal stricture, and two (15.4%) required gastrostomy placement; the mortality rate was 23.1% [1]. In our case series, the complications observed were a vocal cord paralysis in one case, a tracheoesophageal fistula in two cases in which one patient was lost due to sepsis, and esophageal strictures in seven cases. The case with a tracheoesophageal fistula was a 2-month-old boy who was admitted 40 days after ingesting a battery, which was located in the proximal esophagus, and surgical intervention was required. The other tracheoesophageal fistula case was a 2-month-old girl who was admitted 5 h after ingesting a battery, which was located in the proximal esophagus, and the girl died 4 days after developing a fistula. The case with unilateral vocal cord paralysis was a 39-month-old boy who was admitted 24 h after ingesting a battery, which was located in the midesophagus, and who had a grade IIIA mucosal injury; dilatation was implemented.

A history of treatment-resistant pulmonary infection in the 2-month-old boy in our case series who was admitted 40 days after ingestion is notable given the presence of a recurrent lung infection and coughing despite medical therapy, which should raise suspicion of an esophageal foreign body, even in the absence of a witnessed ingestion event [9]. Our findings support monitoring for respiratory symptoms after removing the battery with a prompt emergent evaluation for vocal cord and tracheal complications, including a tracheoesophageal fistula [1].

Similarly, a likelihood of complications developing and leading to mortality even after removal of the battery has been described in two case reports [8, 26], as a consequence of massive bleeding through an esophageal-aortal fistula at 2 weeks [8] or 18 days [26, 27] after removal of the button battery.

In our case series, none of the cases with grade 0 or grade I mucosal injury required dilatation, but dilatation was needed in three of eight cases with grade IIA-IIB injury and all four cases with grade IIIA-IIIB injury. Hence, our findings emphasize the impact of the initial mucosal injury on the clinical outcome after endoscopic removal of a battery, with a higher likelihood of dilatation intervention in patients who present with a higher-grade mucosal injury, particularly grade III injury. This seems notable given that cases with grade IIIA-IIIB mucosal injury (range, 6–24 h) in our series were admitted to the hospital later than those with grade IIA-IIB (range, 4–8 h) or grade 0-I (range, 3–6 h) mucosal injury.

The length of time that the battery is lodged in the esophagus (duration of exposure) increases the severity of esophageal damage, leading to mucosal ulceration and perforation [9, 28–31]. The risk has been reported to significantly increase for foreign bodies remaining in the same location for more than 24 h [32]. The midesophageal location appeared to be associated with a higher risk of prolonged duration-related complications in our case series, given that two cases with a midesophageal location of the ingested battery were admitted to the hospital 24 h after ingestion and both were determined to have a grade III mucosal injury.

Nevertheless, delayed admission is not a definite predictor of poorer outcome in cases of button battery ingestion, as severe injuries may also occur in cases admitted early and diagnosed rapidly [8, 9, 33]. Notably, grade IIA mucosal injury was already evident and was followed by development of a tracheoesophageal fistula and death in a 2-month-old girl in our case series, despite her admission at 5 h after ingesting a battery. Similarly, in another case series study, grade IIIA or IIIB injuries were reported in patients who were admitted within 6 h of battery ingestion [9], and serious complications after ingesting a button battery have been described in children who arrived at the hospital within a short period after ingestion (1–2.5 h) as well as in children with a more delayed admission [3, 8, 34, 35].

Our findings reveal that the ranges for admission time in boys and girls, battery diameter, and age were similar to the current literature. Hence, the tendency for a greater need for dilatation in boys than in girls in our population seems to be related to the higher rate of grade III mucosal injury in boys than in girls.

Community education and raising awareness are crucial to reduce the incidence of childhood aspiration of foreign bodies, which is a preventable condition [36]. Specific to button battery ingestion, recognition of the increased risk associated with newer-age lithium button batteries is required to spur incorporation of stricter legislation for screw-secured battery compartments in devices and more-secure retail packaging into the preventive strategies, along with efforts to raise community awareness [1, 2].

## 5. Conclusion

The present case series study describing the clinical profiles and outcomes of 17 children who ingested an esophageal button battery revealed male predominance, young patient age, and admission at a median 6 hours after ingestion, with nonspecific symptoms at admission. Our findings support the success of rigid endoscopy for urgent removal of an ingested esophageal button battery, but they also indicate the likelihood of severe and potentially lethal complications even after endoscopic removal of the battery and the need for dilatation in nearly half of the cases. Future large-scale clinical studies addressing long-term outcomes in relation to the time from ingestion to admission, esophageal location of the battery, and the mucosal injury grade are necessary to develop a risk stratification model and a well-defined standardized algorithm for postremoval management of children with moderate to severe esophageal injury from ingesting a button battery.

## Figures and Tables

**Table 1:** Overall characteristics (*n*=17).

*Age (months), median (range)*		29 (2–99)
*Gender, n (%)*	Boy	11 (64.7)
	Girl	6 (35.3)
*Symptoms on admission, n (%)*	Hypersalivation	6 (35.3)
	Vomiting + hypersalivation	5 (29.4)
	Dysphagia	2 (11.8)
	Dysphagia + vomiting	2 (11.8)
	Coughing + hypersalivation	1 (5.9)
	Recurrent pulmonary infection + fever	1 (5.9)
*Witness situation, n (%)*	Witnessed	7 (41.2)
	Not witnessed	8 (47.1)
*Location, n (%)*	Proximal esophagus	10 (58.8)
	Midesophagus	3 (17.6)
	Distal esophagus	4 (23.5)
*Time from ingestion to admission (h), median (range) (n*=13)		6 (3–24)
*Diameter of the battery (mm), median (range) (n*=13)		18 (14–22)
*Zargar mucosal injury grade, n (%)*	0	2 (11.8)
	I	2 (11.8)
	IIA	6 (35.3)
	IIB	2 (11.8)
	IIIA	2 (11.8)
	IIIB	2 (11.8)
	Unknown	1 (5.9)
*Postoperative complications, n (%)*		9 (53)
	Early complication-vocal cord paralysis	1 (5.9)
	Late complication-stricture	7 (41.2)
	Late complication-surgery	1 (5.9)

**Table 2:** Case-based characteristics.

Case number	Age (month)	Gender	Location	Symptom	Time from ingestion to admission	Diameter of battery (mm)	Ingestion witnessed	Mucosal injury (Zargar's grade)	Complication	Follow-up
**1**	2	Boy	Proximal	Recurrent pulmonary infection, fever	40 days	UN	No	UN	Tracheoesophageal fistula	Surgery
**2**	77	Boy	Proximal	Hypersalivation	4 h	21	No	I		Normal
**3**	17	Boy	Distal	Dysphagia	6 h	19	Yes	IIA		Normal
**4**	14	Boy	Mid	Vomiting + hypersalivation	UN^*∗*^	18	No	I		Normal
**5**	39	Boy	Proximal	Coughing + hypersalivation	6 h	17	Yes	IIB	Stricture	Dilatation
**6**	28	Girl	Distal	Dysphagia + vomiting	4 h	18	UN	IIA	Stricture	Dilatation
**7**	34	Girl	Distal	Vomiting + hypersalivation	UN	19	Yes	IIA		Normal
**8**	39	Boy	Mid	Dysphagia	24 h	UN	No	IIIA	Unilateral vocal cord paralysis, stricture	Dilatation
**9**	99	Girl	Proximal	Hypersalivation	6 h	20	No	0		Normal
**10**	36	Boy	Proximal	Vomiting + hypersalivation	6 h	17	Yes	IIB		Normal
**11**	2	Girl	Proximal	Hypersalivation	5 h	14	UN	IIA	Tracheoesophageal fistula death after 4 days, sepsis	Death
**12**	8	Boy	Proximal	Hypersalivation	8 h	15	No	IIA		Normal
**13**	42	Girl	Proximal	Hypersalivation	3 h	22	Yes	0		Normal
**14**	55	Girl	Mid	Vomiting + hypersalivation	24 h	19	No	IIIB	Stricture	Dilatation
**15**	15	Boy	Distal	Dysphagia + vomiting	4 h	UN	No	IIA	Stricture	Dilatation
**16**	23	Boy	Proximal	Hypersalivation	UN	18	Yes	IIIB	Stricture	Dilatation
**17**	29	Boy	Proximal	Vomiting + hypersalivation	6 h	UN	Yes	IIIA	Stricture	Dilatation

^*∗*^UN (Unknown).

## Data Availability

The data used to support the findings of this study are included within the article.
